# Assessing the robustness of dose distributions in carbon ion prostate radiotherapy using a fast dose evaluation system

**DOI:** 10.1002/acm2.14528

**Published:** 2024-10-22

**Authors:** Toshiro Tsubouchi, Hiroya Shiomi, Osamu Suzuki, Noriaki Hamatani, Masaaki Takashina, Masashi Yagi, Yushi Wakisaka, Atsuhiro Ogawa, Ayumi Terasawa, Yuichi Akino, Kazuhiko Ogawa, Tatsuaki Kanai

**Affiliations:** ^1^ Department of Medical Physics Osaka Heavy Ion Therapy Center Osaka Japan; ^2^ Department of Radiation Oncology Osaka University Graduate School of Medicine Osaka Japan; ^3^ RADLab Inc. Osaka Japan; ^4^ Department of Carbon Ion Radiotherapy Osaka University Graduate School of Medicine Osaka Japan

**Keywords:** adoptive therapy, bsPTV, carbon ion radiotherapy, fast dose calculation

## Abstract

**Purpose:**

We developed a software program for swiftly calculating dose distributions for carbon ion beams. This study aims to evaluate the accuracy of dose calculations using this software and assess the robustness of dose distribution in treating prostate cancer.

**Methods:**

At the Osaka Heavy Ion Therapy Center, markers are inserted into the prostate gland and used for position verification. To account for geometric changes along the beam path due to marker translation, a beam‐specific planning target volume (bsPTV) is set for each beam. To validate the accuracy of the dose calculations using the developed software, dose distributions for prostate and sarcoma cases were calculated and compared with the treatment planning system. To assess the robustness of the dose distribution, position verification data from 346 cases were utilized to reproduce dose distributions for three matching methods: bone matching, widely adopted in most particle therapy centers; marker translation, which involves direct translation to markers without bone matching; and marker translation after bone matching. The coverage of the target (*D*_99_ of clinical target volume (CTV)) was assessed to evaluate the robustness of the dose distribution. Additionally, statistical analyses were conducted for the dose distributions of each matching method.

**Results:**

The dose calculation for a single condition can be completed very quickly. Statistical analysis revealed significant differences among dose distributions considering the three matching methods. When irradiation was performed with bone matching only, the *D*_99_ was reduced by more than 10% in approximately 7.5% of cases, making it as the poorest among the three matching methods. However, there was no significant reduction in target coverage with the other two methods.

**Conclusion:**

We have demonstrated the accuracy of the developed software for rapidly calculating dose distributions for carbon ion beams and confirmed the robustness of the dose distributions based on the bsPTV.

## 1 INTRODUCTION

Radiation therapy using carbon ions, a type of heavy ions, has garnered significant attention in recent years. The Osaka Heavy Ion Medical Accelerator in Kansai (Osaka HIMAK, HyBeat Heavy‐ion Therapy System, Hitachi, Ltd., Tokyo, Japan) is the sixth carbon ion radiotherapy facility built in Japan, with a total of seven carbon ion radiotherapy facilities in the country.^1^ Carbon ions exhibit a unique physical dose distribution characterized by low lateral scattering and a substantial release of energy at the end of their range, known as the Bragg Peak. Furthermore, their biological effects surpass those of X‐rays, making them highly attractive.^2^, ^3^, ^4^ Osaka HIMAK currently administers over 1000 cases per year, with prostate cancer constituting approximately 70% of all treated cases.

Carbon ion radiation therapy (CIRT) for prostate cancer is commonly administered at Osaka HIMAK using a prescribed dose of 51.6 Gy (RBE) delivered in 12 fractions, which is relatively fewer fractions compared to X‐rays and protons.^5^, ^6^ To ensure accurate irradiation of the prostate, one or two gold markers (Gold Anchor, Naslund Medical AB, Huddinge, Sweden) inserted into the prostate are used for position matching, utilizing frontal and lateral radiographs.^7^ The number of markers is less than three, so rotational corrections using markers are not performed. In our center, the position verification method for prostate patients involves only marker translation after bone matching. For patients who cannot have markers inserted into their prostate for any reason, we have an in‐room computed tomography (CT) in one of our three treatment rooms. This in‐room CT is used to perform prostate translation after bone matching.

Particle beams, due to their unique physical property of Bragg peaks, require accurate range estimation. At Osaka HIMAK, the stoichiometric calibration method proposed by Schneider is utilized to estimate the range from CT values and the uncertainties in the range estimation of patient stopping power ratio (SPR) are addressed by applying a 3.5% margin at both distal and proximal ends of target volume to ensure comprehensive target coverage across different tumor sites.^8^, ^9^, ^10^ Carbon ions are highly sensitive to geometric and anatomical changes along the beam path. While the latter is currently unaccounted for, geometric changes resulting from the matching method are managed by defining a beam‐specific planning target volume (bsPTV) for each beam.^11^ The bsPTV is calculated and applied to the clinical target volume (CTV) based on the concept of pre‐expanding the CTV laterally to account for geometric changes along the beam path occurring within that expanded region. This ensures dose coverage to the target even in the presence of such geometric variations.^7^, ^11^

Consequently, the treatment plan is meticulously designed to compensate for geometric variations resulting from setup errors and range uncertainties arising from the conversion of CT values to relative stopping power. This ensures a highly robust dose distribution. However, the quantification of the actual extent of dose distribution robustness remains unexplored due to the time‐consuming nature of dose calculations that consider errors in the six‐axis components. To address this, a system capable of rapidly calculating dose distributions within a few seconds has been developed. This system reproduces the dose distribution on the CT images by deforming the dose distribution in water calculated in the treatment planning system (TPS), based on the relative stopping power to water (RSP) observed on the CT images. This allows for a quicker reproduction of dose distribution compared to actual dose calculations. In essence, the crucial aspect is how precisely the developed software reproduces the dose distribution of the TPS. In this study, we first validated the calculation accuracy of our software through a comparison with the TPS. Our software is not designed as a secondary check for dose calculations; instead, its primary function is to faithfully replicate the TPS's dose distribution and swiftly assess the robustness of that distribution.

With this system, we have conducted an evaluation of the dose distribution's robustness using clinically acquired setup data.

## 2 MATERIALS AND METHODS

This study was approved by an institutional review board.

### 2.1 Treatment planning of CIRT for prostates

**FIGURE 1:**
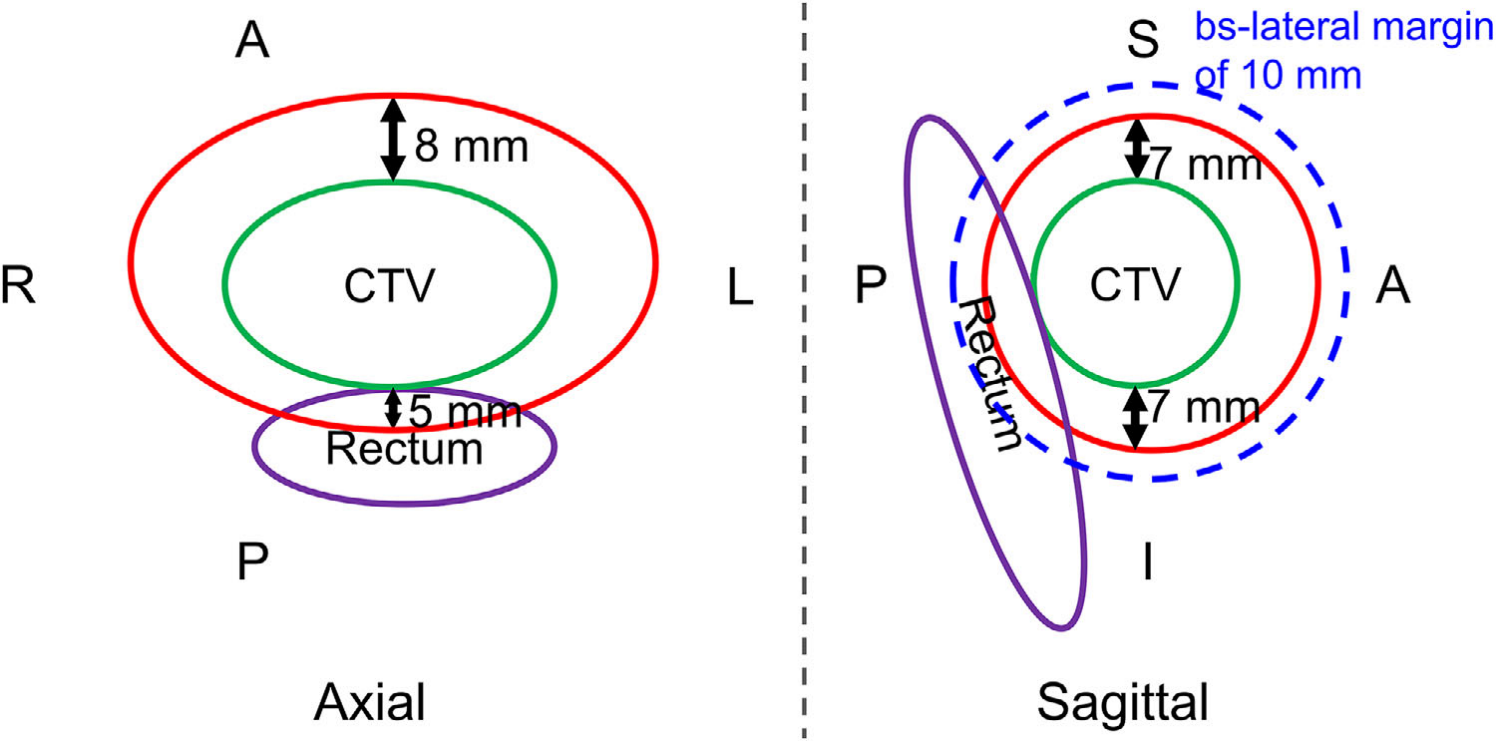
The diagram illustrates an overview of the margin in a prostate treatment plan. The red‐outlined region represents the irradiation field, with margins set at 7 mm in the S (superior), 7 mm in the I (inferior), 8 mm in the A (anterior), and 5 mm in the P (posterior) directions with respect to the clinical target volume (CTV) when viewed from the beam. The bs‐lateral margin, indicated by blue dashed lines, is set at 10 mm laterally in the direction of the beam as a searching area.

Prostate treatment with carbon ion beams involves irradiation using opposed lateral beams over 12 fractions, with a prescribed dose of 51.6 Gy (RBE). Details regarding dose constraints for the target and organs at risk (OARs), as well as specifics related to bsPTV, are documented in a previously published paper.^7^ Here, I will briefly discuss the margin settings.^7^ For the prostate treatment plan, margins of 5 mm posteriorly, 8 mm anteriorly, and 7 mm superiorly and inferiorly are applied with respect to the CTV, which corresponds to the prostate, as shown in Figure 1.^7^ These margins account for the potential movement of the prostate during irradiation. In terms of the beam direction, a 3.5% margin is set on both the proximal and distal sides of the target to accommodate range uncertainties.^9^ Furthermore, a bs‐lateral margin of 10 mm is implemented to compensate for geometric changes along the beam path. The bs‐lateral margin allows for a theoretical translation of up to 10 mm to the marker.^7^

### 2.2 Fast dose calculation software

The ShioRIS2 particle edition (SP2) software, developed by RadLab Inc. in Osaka, Japan, has the capability to efficiently calculate dose distributions for carbon beams within the body. This software considers setup errors and the geometric conditions of the beam that directly influence the dose distribution. By conducting the study, we successfully showcased the computational accuracy of the SP2 software, ensuring reliable dose calculations. Furthermore, we assessed the robustness of the dose distributions by incorporating clinically obtained data on prostate motion. This evaluation provides valuable insights into the reliability and stability of the treatment planning process for prostate cancer using the SP2 software.

#### 2.2.1 Dose calculation method in SP2

In particle therapy, carbon and proton beams have a higher biological effect compared to X‐rays, necessitating the calculation of clinical doses considering the relative biological effectiveness (RBE). At our center, we use the VQA treatment planning system (VQA, Hitachi, Ltd., Japan) for dose optimization and calculation. The physical dose is calculated using a pencil beam triple Gaussian model. We adopted the mixed beam model, based on the linear quadratic (LQ) model and the theory of mixed radiation fields, to estimate the biological effect. In the mixed beam model, the RBE giving a 10% survival fraction of human salivary gland tumor (HSG) cells is fixed without dose dependency to evaluate the clinical dose. Details on the dose calculation algorithm are described in our previous publications.^9^, ^12^ In order to rapidly calculate the dose distribution while accounting for setup errors, the SP2 software employs a method that does not directly calculate the dose from the carbon ion spot positions and dose per spot, as the VQA dose. Instead, it reproduces the dose distribution on CT images by transforming the dose distribution in water calculated using the VQA.^9^, ^12^ This approach enables the dose calculation process to be completed in a matter of seconds because it focuses on each beam ray direction and does not account for the lateral direction. Here, it is important to note that all dose distributions calculated by SP2 and VQA presented hereafter are clinical dose distributions.

**FIGURE 2:**
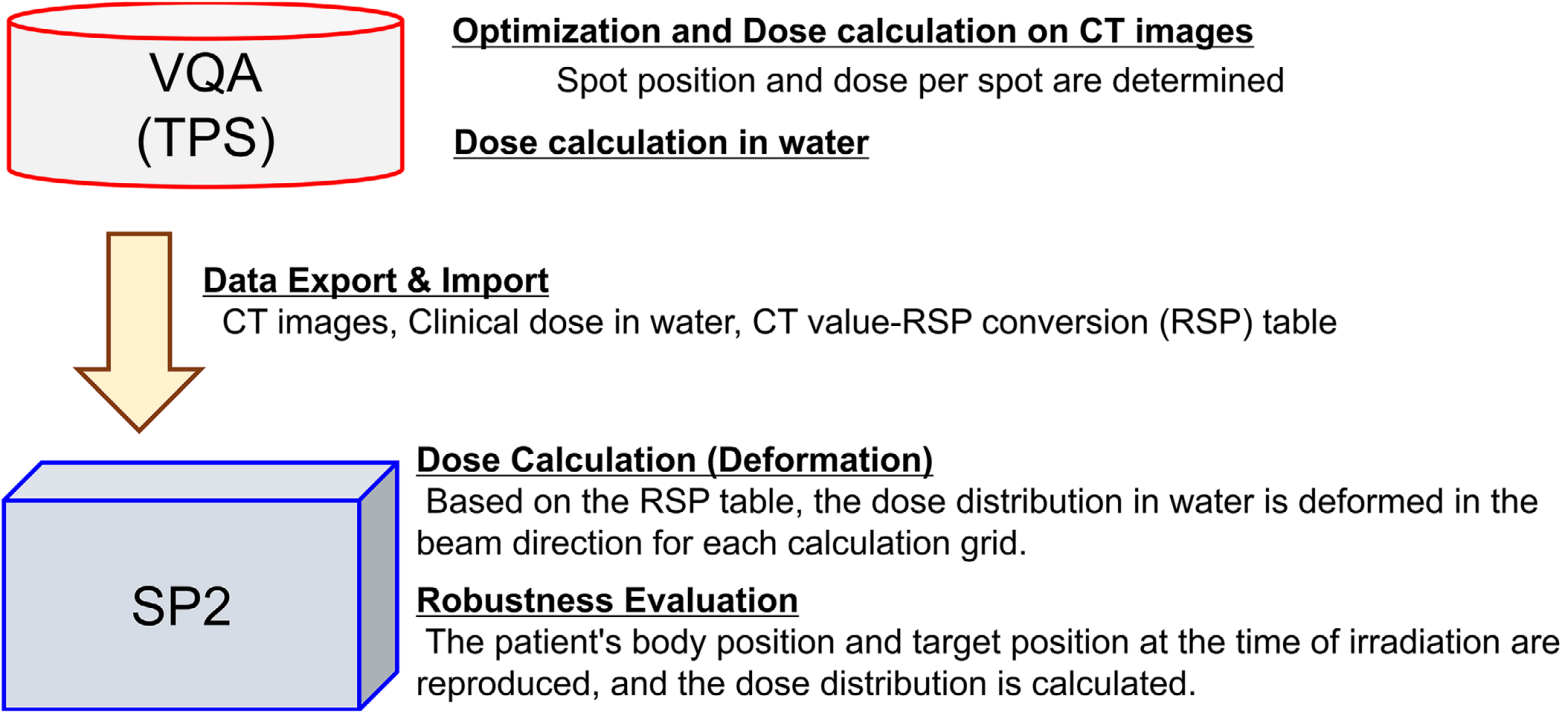
Schematic representation of SP2 dose calculation process: SP2 imports the dose distribution in water calculated by the TPS and transforms it based on the CT‐relative stopping power to water conversion table to reproduce the dose distribution on CT images. This facilitates rapid dose evaluation on CT images. TPS, treatment planning system.

To illustrate this conversion process, Figure 2 provides a visual representation of how the dose distribution in water, obtained from VQA, is transformed into the dose distribution on CT images using the SP2 software.

**FIGURE 3:**
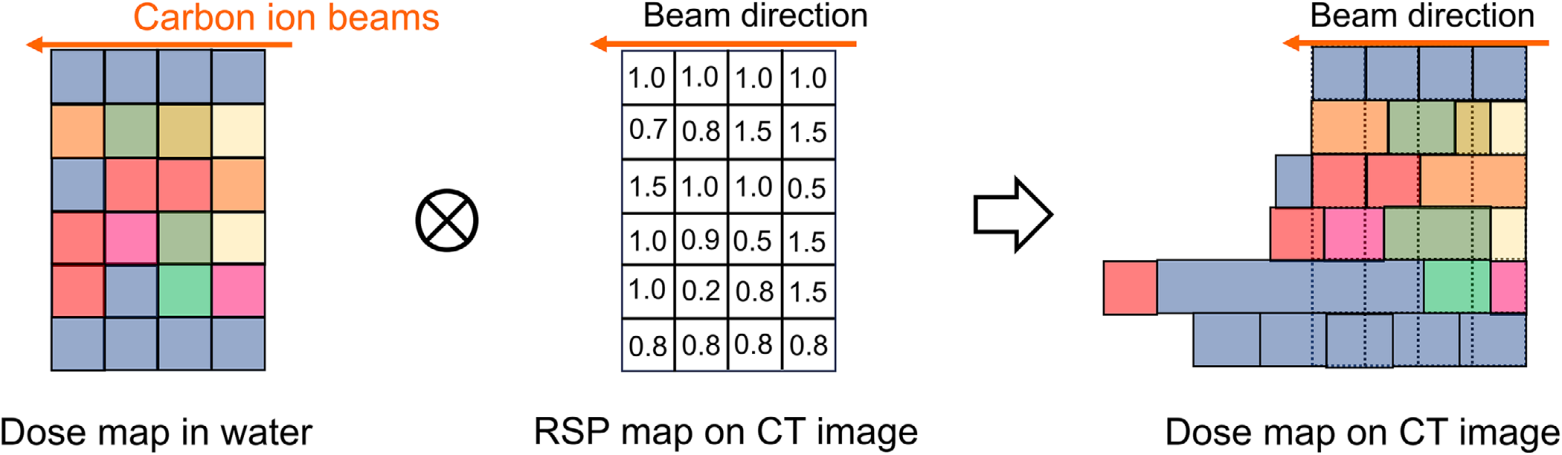
Conceptual diagram illustrating the process of generating dose distributions on CT images using SP2. SP2, ShioRIS2 particle edition.

The process of converting the dose distribution calculated by VQA into the dose distribution on CT images using the SP2 software is as follows:

1. The VQA system performs optimization on the CT images to determine the spot positions and their corresponding doses, resulting in the calculation of the dose distribution in water.
2. SP2 imports the necessary data for the calculation, including dose distribution in water calculated by VQA and CT images of the patient for dose calculation in SP2. Additionally, a conversion table between CT values and RSP, known as the CT‐RSP conversion table, is utilized.
3. Using the CT‐RSP conversion table, SP2 first converts the CT images to an RSP map. Subsequently, the computational grid that contains the dose data is deformed (or stretched) in the beam direction based on the RSP values to reproduce the dose distribution on the CT images (Figure 3).
4. When calculating the dose distribution considering setup errors, the geometric relationship with the target, accounting for setup errors, is represented by adjusting the beam according to the setup errors relative to the target. Subsequent dose calculations follow the approach in (3), deforming the dose distribution in water based on the RSP along the beam direction.

This process ensures that the dose distribution calculated by SP2 accurately corresponds to the patient's anatomy by considering the CT‐RSP conversion and deformations in the computational grid. To validate the accuracy of SP2 calculations, we conducted a study using data from 10 randomly selected prostate patients. Using SP2, we reproduced the dose distributions for these patients and compared them with the dose distributions obtained from VQA.

#### 2.2.2 Dose calculation accuracy of SP2

In this study, the robustness assessment of dose distribution is conducted using Planning CT. However, for practical clinical application, specifically evaluating dose distribution on day‐to‐day CT images, it is crucial to verify whether the accuracy of SP2 calculations is maintained across different CT datasets. Patients undergoing CIRT with in‐room CT position verification have 12 CT datasets. Therefore, using different CT images from the same patient, dose distributions can be computed on each CT using SP2 and compared with VQA.

Additionally, while medium (208.3–329.6 MeV/u) and high (329.6–430.0 MeV/u) energy ranges, from a total of 12 accelerated energies (ranging from 100.0 to 430.0 MeV/u), were used in prostate treatments, the accuracy of dose distribution, including the low‐energy range (100.0–208.3 MeV/u), was verified using a sarcoma case where the tumor is located superficially.

#### 2.2.3 Robustness evaluation of dose distribution

SP2 has the capability to calculate the dose distribution while considering setup errors and potential range errors that may occur during treatment. It assesses the robustness of the dose distribution by analyzing the coverage of the target in the resulting distribution. To evaluate the robustness, patient position verification data from 346 prostate patients treated at Osaka HIMAK were utilized. The position verification data were recorded for each irradiation, resulting in a total of 346 × 12 (number of patients x number of fractions) data points. For 10 prostate cases, dose distributions were calculated, taking into account setup errors from 346 × 12 sessions mentioned above, and the robustness of the dose distribution was evaluated based on the coverage of the target for each case in the following formula.

(1) $$\mathrm{Robustness}\;=\;\frac{D_{99}\left(\mathrm{Error}\right)}{D_{99}\left(\mathrm{Original}\right)}$$

Here, *D*_99_(Original) represents the *D*_99_ for the dose distribution without errors, and *D*_99_(Error) represents the *D*_99_ of CTV for the dose distribution calculated considering errors.

In this evaluation, we assessed the ability of the dose distribution to cover the target using different patient matching methods: bone‐only (Bone+, Marker−), marker‐only (Bone−, Marker+), and marker translation after bone matching (Bone+, Marker+). The following describes the positional relationship between the beam and the target for each matching method.:

1. Bone+, Marker−: In this method, only the rotational component is corrected during bone matching, while the beam relative to the target is not adjusted. Essentially, the beam aligns with the bone, but the target shifts based on the bone‐to‐marker displacement.
2. Bone−, Marker+: This method skips bone matching and directly translates to the marker position. The positional relationship between the beam and the bone is disregarded, and the beam relative to the target is adjusted. In other words, the rotational component of the beam relative to the bone is ignored during dose calculation.
3. Bone+, Marker+: This patient matching method involves performing marker translation after bone matching. The dose calculation replicates a state where the rotational component of the beam relative to the bone and the positional relationship between the target and the beam are all aligned.

In the context of prostate treatment, the first method (Bone+, Marker−) represents a matching approach widely employed in many particle therapy centers in Japan.^5^, ^13^ The third method (Bone+, Marker+) corresponds to the matching technique implemented at our center. As for the second method (Bone−, Marker+), it represents a simplified version of our center's matching approach. In this case, bone matching is skipped, and the beam directly translates to the marker position. Dose distributions were calculated for each of these three methods, and their robustness was evaluated. The robustness of dose distribution among three position verification methods was also assessed for statistical differences using either a two‐tailed paired *t*‐test or a Wilcoxon signed‐rank test, depending on the outcomes of the Shapiro–Wilk test. Statistical significance was considered at *p* <  0.05. Additionally, dose distributions were also calculated and evaluated for robustness under a range change of ±3.5%.

## 3 RESULTS

### 3.1 Dose calculation accuracy of SP2

**FIGURE 4:**
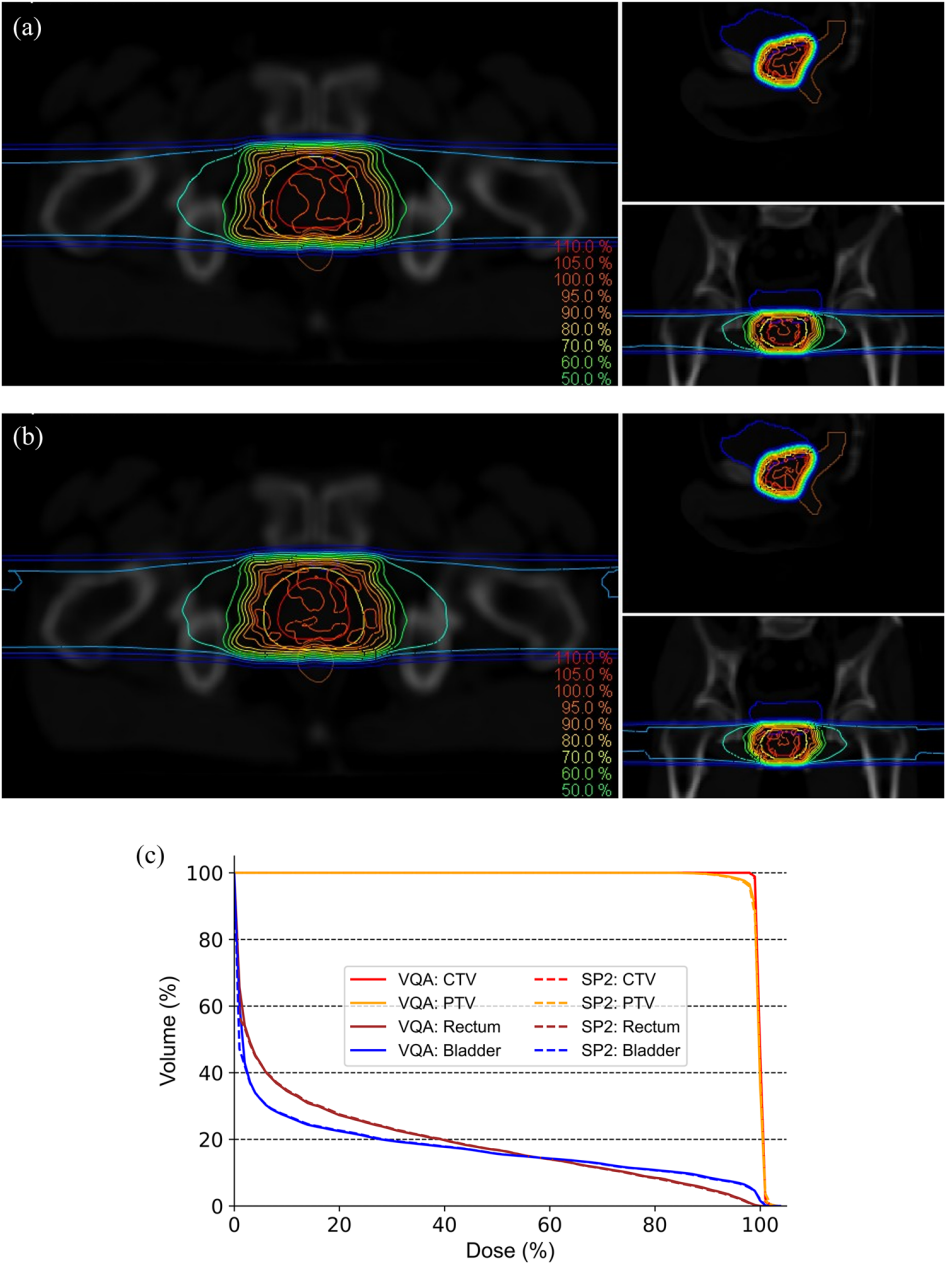
Dose distributions calculated with VQA (a) and SP2 (b) are shown. (c) shows the DVH obtained from VQA and SP2 for a prostate case.

**FIGURE 5:**
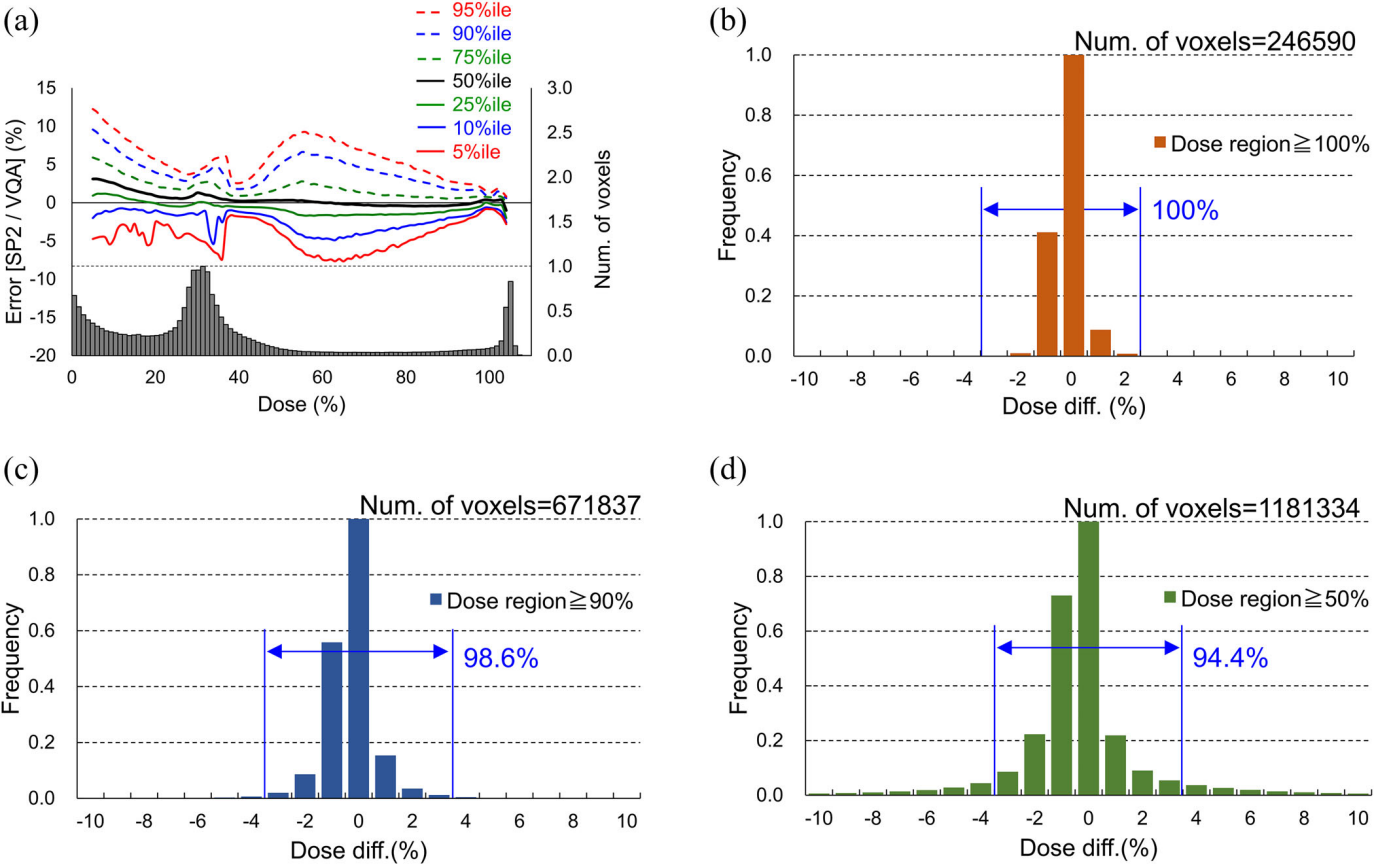
The results of dose distributions on CT images calculated by SP2 and VQA for the 10 prostate cases are presented. In (a), a voxel‐by‐voxel comparison of the data from the 10 cases is performed, and the differences are displayed as a function of dose. The histogram at the bottom of (a) illustrates the relative distribution of voxel counts across different doses. The results demonstrate excellent agreement in the high‐dose region, corresponding to the target area, while revealing relatively weaker agreement in the medium‐ and low‐dose regions. (b)–(d) depict the comparison results for dose regions at or above 100%, 90%, and 50% of the prescribed dose, respectively, revealing that the discrepancy between SP2 and VQA becomes more pronounced when the low‐dose range is included.

**FIGURE 6:**
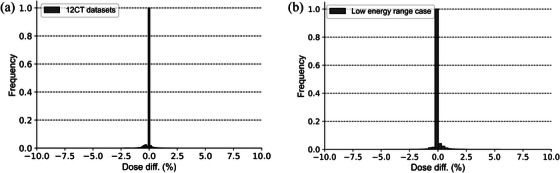
(a) A histogram illustrates the difference between the dose calculation results of SP2 and VQA across 12 different CT scans of the same prostate case, highlighting good agreement with VQA on other than the planning CT. (b) The accuracy of dose distribution calculations, including the low‐energy range, aligns well with VQA, indicating the calculation precision independent of the energy range.

Figure 4 illustrates the dose distributions for VQA (a) and SP2 (b), respectively. Additionally, it shows the comparison of the dose‐volume histogram (DVH) for a prostate case (c), demonstrating good agreement between the two calculation methods. Figure 5(a) displays a comparison of the dose distributions calculated by VQA and SP2 for the 10 prostate cases. This figure illustrates the voxel‐by‐voxel differences between SP2 and VQA, with a histogram at the bottom representing the relative distribution of voxel counts corresponding to each dose. It is observed that the differences in dose distribution between VQA and SP2 are initially smaller in the middle dose range. However, these differences become more significant in the low dose range. Notably, in the high dose range, the dose distributions are in good agreement with each other. To further analyze the dose differences, Figure 5(b)–(d) present histograms focusing on specific areas: doses above 100%, 90%, and 50% of the prescribed dose, respectively. These histograms provide insights into the error distribution for the dose differences observed in Figure 5(a). The results reveal that the dose distribution in the target area exhibits excellent agreement between VQA and SP2. In Figure 6(a), the results depict the comparison of dose distributions calculated using VQA and SP2 on daily CT scans. The histogram illustrates the differences between VQA and SP2, revealing minimal discrepancies. This suggests that the reproducibility of dose distribution with SP2 extends to CT scans beyond the Planning CT. Figure 6(b) displays the histogram of differences between SP2 and VQA in dose distribution calculations for the sarcoma case, specifically in the low‐energy range. Similarly, minimal differences were observed, affirming the accuracy of SP2 in calculating dose distributions across all energy ranges. These findings highlight the calculation accuracy of SP2, which transforms the dose distribution in water to reproduce the dose distribution on CT images based on the RSP map.

### 3.2 Robustness of prostate treatment planning dose distribution

**FIGURE 7:**
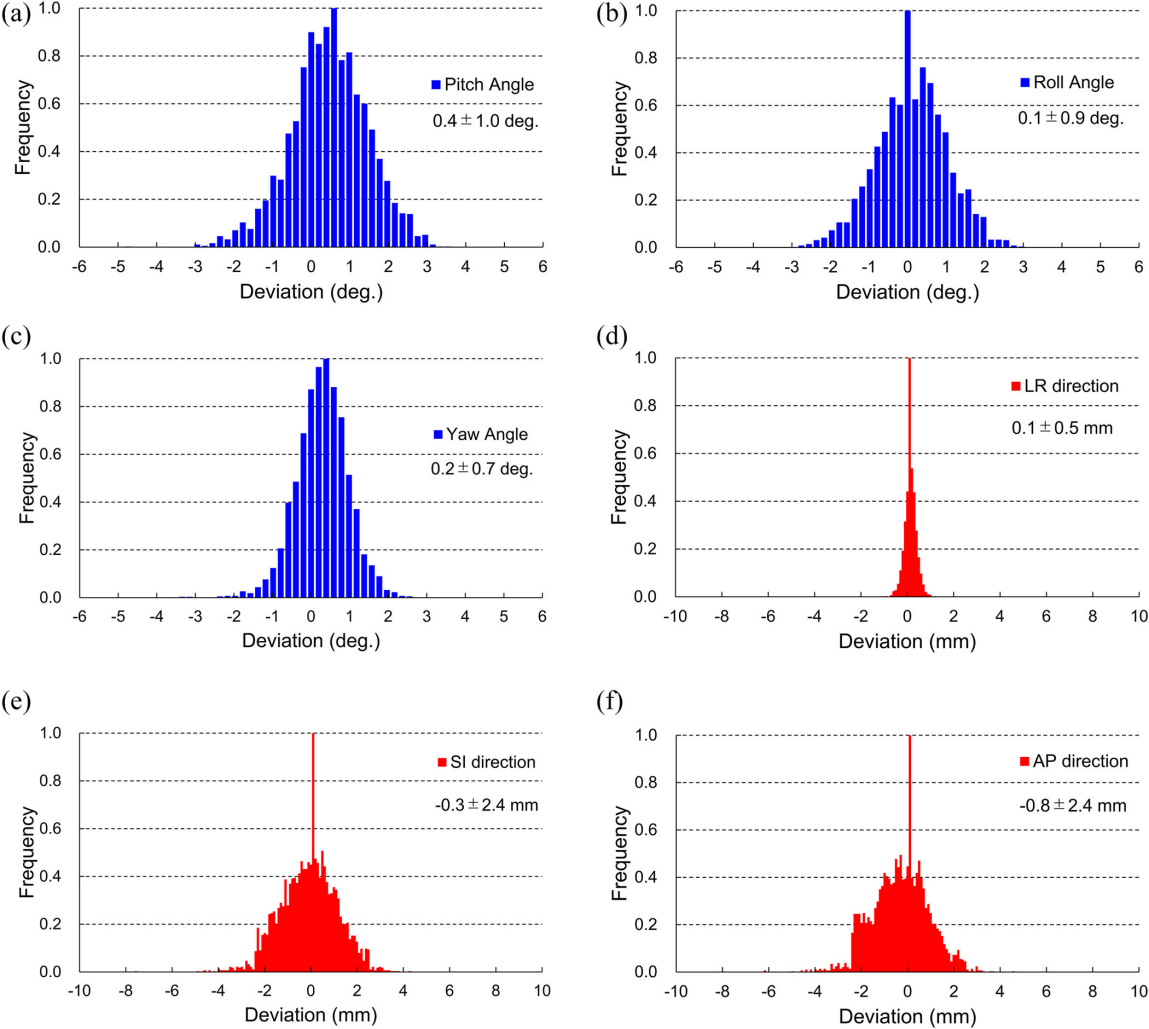
Patient position matching data obtained from 346 prostate patients are presented. The figure displays angular correction data obtained from bone matching as well as the amount of translation to the marker.

Figure 7 provides a summary of position verification data per fraction for 346 prostate cases. This data includes the amount of angular correction achieved through bone matching and the amount of translation to the marker. The positional relationship between the beam and the target for each irradiation was reproduced based on this data, and the dose distribution was simulated accordingly.

**FIGURE 8:**
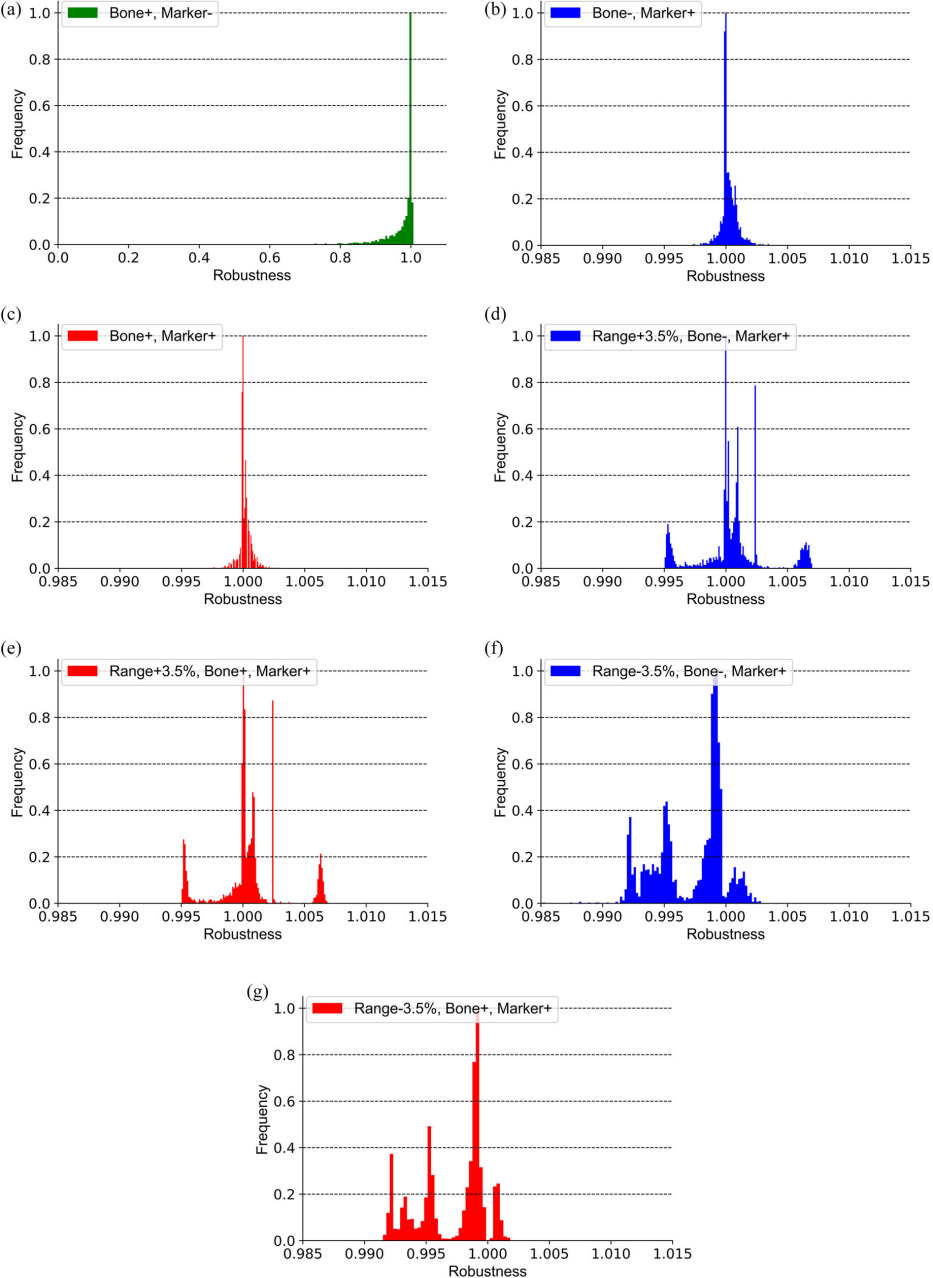
The target coverage of the dose distribution is depicted for different matching methods: bone matching only (a), marker translation only (b), and a combination of both (c). The *X*‐axis represents the degree of agreement with the original dose distribution for the target *D*_99_. The target coverage for bone matching alone is lower compared to other matching methods, as it does not account for the displacement between the beam and target. Conversely, for marker translation and marker translation after bone matching, the dose distribution is calculated with range uncertainty. As depicted in (d)–(g), it can be observed that the target coverage remains consistently high in both cases. *D*_99_: dose to 99% of the volume.

Figure 8 illustrates the results of robust evaluation, with the horizontal axis representing the agreement (*D*_99_) calculated using Equation (1) between the original dose distribution and the dose distribution calculated while considering the setup error.

A value of 1.0 indicates a perfect match with the original distribution. Figure 8 presents the results of dose calculations and comparisons with the original dose distributions for the three matching methods described in section 2.2.2. Specifically, (a)–(c) correspond to the dose calculations for these matching methods. Additionally, (d)–(g) show the comparison results for the “Bone−, Marker+” and “Bone+, Marker+” methods, taking into account a range error of ±3.5%.

Tables 1 and 2 summarize the data presented in Figure 8. The results demonstrate that dose coverage of the target is inferior with bone matching alone compared to other position matching methods. However, statistical analysis of the target coverage for dose distributions considering errors revealed significant differences in each comparison (*p*‐values were nearly 0 in each comparison). In the case of “Bone−, Marker+” and “Bone+, Marker+” methods, the dose administration to the target is well assured, even when considering range uncertainties of ±3.5%.

**TABLE 1:** The target coverage of the dose distribution for each matching method is presented. Bone matching alone leads to a 10% reduction in the *D*_99_ of the target compared to the original treatment plan, equivalent to approximately 7.5%.

Decrement in *D*_99_ (%)	Bone+, Marker− (%)	Bone−, Marker+ (%)	Bone+, Marker+ (%)
1	39.5	0	0
5	17.8	0	0
10	7.5	0	0

**TABLE 2:** The results of evaluating the target coverage of the dose distribution, considering a range uncertainty of 3.5% for the matching method other than bone matching, are presented below. The absence of positional displacement of the target and beam through marker translation (marker matching) ensures adequate dose coverage with no issues.

	Range error: +3.5 (%)		Range error: −3.5 (%)	
Decrement in *D*_99_ (%)	Bone‐, Marker+	Bone+, Marker+	Bone‐, Marker+	Bone+, Marker+
1	0	0	0.29	0.087
5	0	0	0	0
10	0	0	0	0

## 4 DISCUSSION

The SP2 software was developed with the aim of rapidly reproducing the dose distribution on CT images by deforming the dose distribution in water along the beam direction according to the RSP. In the case of 10 randomly selected cases, the dose distributions calculated by SP2 exhibited a close correspondence with those computed by VQA, underscoring the high precision of the dose calculation. Additionally, when considering cases with targets located relatively superficially, the dose calculations performed by SP2 consistently matched the results obtained through VQA. This result indicates a good agreement with VQA, irrespective of the energy employed. Moreover, to assess the reproducibility when performing dose calculations on CT images other than the Planning CT, 12 different CT images of the same patient were used to conduct dose calculations, confirming the consistency of results across multiple imaging scenarios.

As shown in Figure 5(a), it was observed that the calculation accuracy decreases in the low dose region compared to the high dose region. The low dose region is mainly characterized by two elements: the secondary particle components occurring in the distal part behind the Bragg peak and the scattering components in the lateral region. Regarding the former, as it occurs along the beam direction, SP2 can reproduce it, but considering the lateral components in SP2 is challenging due to its calculation principle of deforming dose distribution based on RSP along the beam direction. However, the calculation accuracy in the middle to high dose range is good, and overall, it reproduces the dose distribution well. Utilizing the SP2 software, we further calculated the dose distribution based on position verification data from 346 cases at our center. By accurately reproducing the positional relationship between the beam and the target during treatment, we were able to evaluate the coverage of the target and confirm the robustness of the dose distribution. In addition to accounting for setup errors, we conducted dose calculations considering a range uncertainty of ±3.5%. The results demonstrated that the dose distribution adequately covered the target even in the presence of such range uncertainties. These findings underscore the ability of the SP2 software to accurately calculate dose distributions while accounting for various uncertainties, such as setup errors and range uncertainties.

To account for uncertainties related to the CT image and CT‐RSP conversion table, as well as to accommodate geometric changes on the beam path, we implemented a 3.5% range margin in the beam direction. This range margin, along with the use of bsPTV to handle marker translation‐induced geometric changes, are the key factors contributing to the robustness of the dose distribution.^11^ In our evaluation, we assessed the impact of different matching methods on the target coverage of the dose distribution. Interestingly, although the dose distributions considering each matching method exhibited statistically significant differences from one another, we found no significant difference of target coverage between direct marker translation and marker translation after bone matching, shown in Table 1. This suggests that both matching methods adequately cover the target in terms of dose distribution. Consequently, it is clinically acceptable to perform marker translation without bone matching or after a less precise bone matching procedure. The excellent robustness of the dose distribution can be attributed to the bsPTV, which replaces all water‐equivalent lengths in the margin with the maximum range obtained through ray tracing within the specified search range (bs‐lateral). According to the previous papers, in carbon ion prostate therapy delivered with bilateral irradiation, it has been reported that marker translation (marker matching) not only ensures dose coverage to the target but also helps maintain lower doses to nearby organs at risk (OARs), such as the rectum and bladder, as expected.^14^, ^15^ On the other hand, it is important to note that it may inadvertently increase the dose to OARs along the beam direction. Based on our study and previous reports, as long as marker matching is performed for prostate cases, the rectum dose and target dose coverage can be maintained as expected in the treatment plan. However, in clinical practice, if the amount of marker translation exceeds 5.0 mm and this occurs twice within the initial six fractions, we conduct CT simulations to confirm whether the rectum dose and dose coverage are acceptable. If the evaluation results exceed the criteria, the treatment plans need to be recreated. As for the irradiation performed with only bone matching, we observed significantly worse target coverage compared to other matching methods. This underscores the criticality of correcting the displacement from the bone to the marker before irradiation in order to guarantee accurate dose delivery to the prostate.^14^ In summary, our findings highlight the effectiveness of marker matching and the necessity of correcting bone‐to‐marker displacement for ensuring reliable target coverage and dose distribution. The incorporation of the bsPTV, while enhancing target coverage, requires careful consideration of the potential impact on OARs.

There are several limitations to be acknowledged in this study. Firstly, the ability of SP2 to replicate the dose distribution of the TPS on CT images other than the planning CT images has been verified. However, the robust evaluation has been conducted only on the planning CT images, which means that the robustness study did not account for daily anatomical changes that may affect the dose distribution. This limitation prevents the consideration of the impact of these changes on the robustness of the dose distribution. Secondly, the evaluation of dose distribution robustness focused solely on target coverage, without considering the dose to OARs. Therefore, the study did not comprehensively assess the potential impact on OARs, which is an important aspect of treatment planning and evaluation. Thirdly, the calculation method employed by SP2, which transforms the dose distribution in water along the beam direction based on the patient's CT‐derived RSP map, may not guarantee accurate calculations in regions with significant inhomogeneities, such as the lung and head and neck sites. Moreover, the simulation did not account for target motion during irradiation (intra‐fractional motion). Although the field size was adjusted to accommodate intra‐fractional motion, the study did not explicitly consider its effects.

Given these limitations, the robustness evaluation of the dose distribution by SP2 proves particularly effective for prostate cases. Moreover, the calculation accuracy with SP2 was verified for each energy range, indicating its applicability to nearly all sites except for the head and neck or lung regions. However, in abdominal cases such as the pancreas, where the state of intestinal gas changes daily, SP2 may face challenges due to these heterogeneous conditions. Nevertheless, we believe SP2 can be applied effectively in sarcoma and liver cases, where there are relatively few heterogeneous regions. If CT images can be obtained immediately before irradiation, combining position matching data from the day with CT image data allows for the prediction of target coverage for the intended beam, potentially serving as a foundation for future particle beam adaptive therapy. It is essential to address these limitations in future studies to provide a more comprehensive assessment of the dose distribution and further enhance treatment planning accuracy and adaptability.

## 5 CONCLUSION

The development of the fast dose calculation system, SP2, was successful, and SP2 accurately reproduces the dose distribution of the treatment planning system, ensuring the accuracy and reliability of its calculations. Additionally, the robustness of the prostate dose distribution used in treatments at Osaka HIMAK was confirmed.

## AUTHOR CONTRIBUTIONS

*Conceptualization*: Toshiro Tsubouchi and Hiroya Shiomi. *Methodology*: Toshiro Tsubouchi, Hiroya Shiomi, Osamu Suzuki, Noriaki Hamatani, Masaaki Takashina, Masashi Yagi, Yushi Wakisaka, Atsuhiro Ogawa, Aumi Terasawa. *Formal analysis*: Toshiro Tsubouchi, Osamu Suzuki, and Hiroya Shiomi. *Data acquisition and analysis*: Toshiro Tsubouchi, Hiroya Shiomi, Noriaki Hamatani, Masaaki Takashina, Masashi Yagi, Yushi Wakisaka, Atsuhiro Ogawa, Ayumi Terasawa. *Data curation*: Hiroya Shiomi. *Writing—original draft preparation*: Toshiro Tsubouchi. *Writing—review and editing*: Toshiro Tsubouchi. *Visualization*: Toshiro Tsubouchi. *Supervision*: Osamu Suzuki, Kazuhiko Ogawa, Tatsuaki Kanai. *Project administration*: Toshiro Tsubouchi, Osamu Suzuki and Hiroya Shiomi. All authors critically revised the report, commented on drafts of the manuscript and approved the final report.

## CONFLICT OF INTEREST STATEMENT

Hiroya Shiomi is a founder of RADLab Inc.

## Data Availability

The data that support the findings of this study are available from the corresponding author upon reasonable request.
